# Distribution of euptyctimous mite *Phthiracarus longulus* (Acari: Oribatida) under future climate change in the Palearctic

**DOI:** 10.1038/s41598-024-72852-8

**Published:** 2024-09-19

**Authors:** Tomasz Marquardt, Sławomir Kaczmarek, Wojciech Niedbała

**Affiliations:** 1 Department of Evolutionary Biology, Faculty of Biological Sciences, Kazimierz Wielki University, Bydgoszcz, Poland; 2 Department of Animal Taxonomy and Ecology, Faculty of Biology, Adam Mickiewicz University, Poznań, Poland

**Keywords:** Acariformes, Species range, Climatic scenario, Environmental niche modelling, Maximum entropy method, Ecology, Animal migration, Biodiversity, Biogeography, Climate-change ecology, Ecological modelling

## Abstract

The aim of this paper is to prepare, describe and discuss the models of the current and future distribution of *Phthiracarus longulus* (Koch, 1841) (Acari: Oribatida: Euptyctima), the oribatid mite species widely distributed within the Palearctic. We used the maximum entropy (MAXENT) method to predict its current and future (until the year 2100) distribution based on macroclimatic bio-variables. To our best knowledge, this is the first-ever prediction of distribution in mite species using environmental niche modelling. The main thermal variables that shape the current distribution of *P. longulus* are the temperature annual range, mean temperature of the coldest quarter and the annual mean temperature, while for precipitation variables the most important is precipitation of the driest quarter. Regardless of the climatic change scenario (SSP1-2.6, SSP2-4.5, SSP5-8.5) our models show generally the northward shift of species range, and in Southern Europe the loss of most habitats with parallel upslope shift. According to our current model, the most of suitable habitats for *P. longulus* are located in the European part of Palearctic. In general, the species range is mostly affected in Europe. The most stable areas of *P. longulus* distribution were the Jutland with surrounding southern coasts of Scandinavia, islands of the Danish Straits and the region of Trondheim Fjord.

**Supplementary Information:**

The online version contains supplementary material available at 10.1038/s41598-024-72852-8.

## Introduction

There is no doubt, that the continuation of emissions will cause the irreversible effects on the climate and the life on Earth^1^. The response of plants and animals is widely studied and modelled at various levels under either natural or experimental conditions^2–14^. The insects are intensively studied and their response to the climate change is of much concern because of provided ecosystem services as e.g. pollination or their importance as pests^2,15,16^. Insects, however, as primarily atmobionts, provide the ecosystem services mainly in the above-ground part of land-habitats. What is important, the above- and below-ground parts of terrestrial ecosystems are interconnected, mutually dependent and influenced by the climate; however, still not fully understood^17–26^. In the below-ground part (i.e. soil), fauna and microbes influence directly and indirectly on the dead organic matter decomposition, the process that is driven by the organic compounds from the above-ground part, and at the same time crucial to maintain the above-ground life^27–30^. The arthropods are numerous in the soil fauna, and in most habitats, the moss mites (Oribatida) are dominant, however, the mite fauna is still poorly studied regarding climatic change^22,31^. Mainly detritivores, oribatids influence on the decomposition processes, but also improve spreading of primary decomposers in the soil profile, i.e. fungi and bacteria. We don’t know, how the climate change will influence on these mites, and how this response will spread within ecosystems. Putting aside this question, the first step is to predict the possible change in the distribution of species due to the climate change. In this work, we focus on the distribution of the Holarctic oribatid mite species: *Phthiracarus longulus* (Koch)^32^ (Oribatida: Euptyctima) within Palearctic using the maximum entropy modelling (MAXENT), a method that wasn’t applied to mite species so far.

## Results

### Model accuracy, variable importance and response

**Fig. 1:**
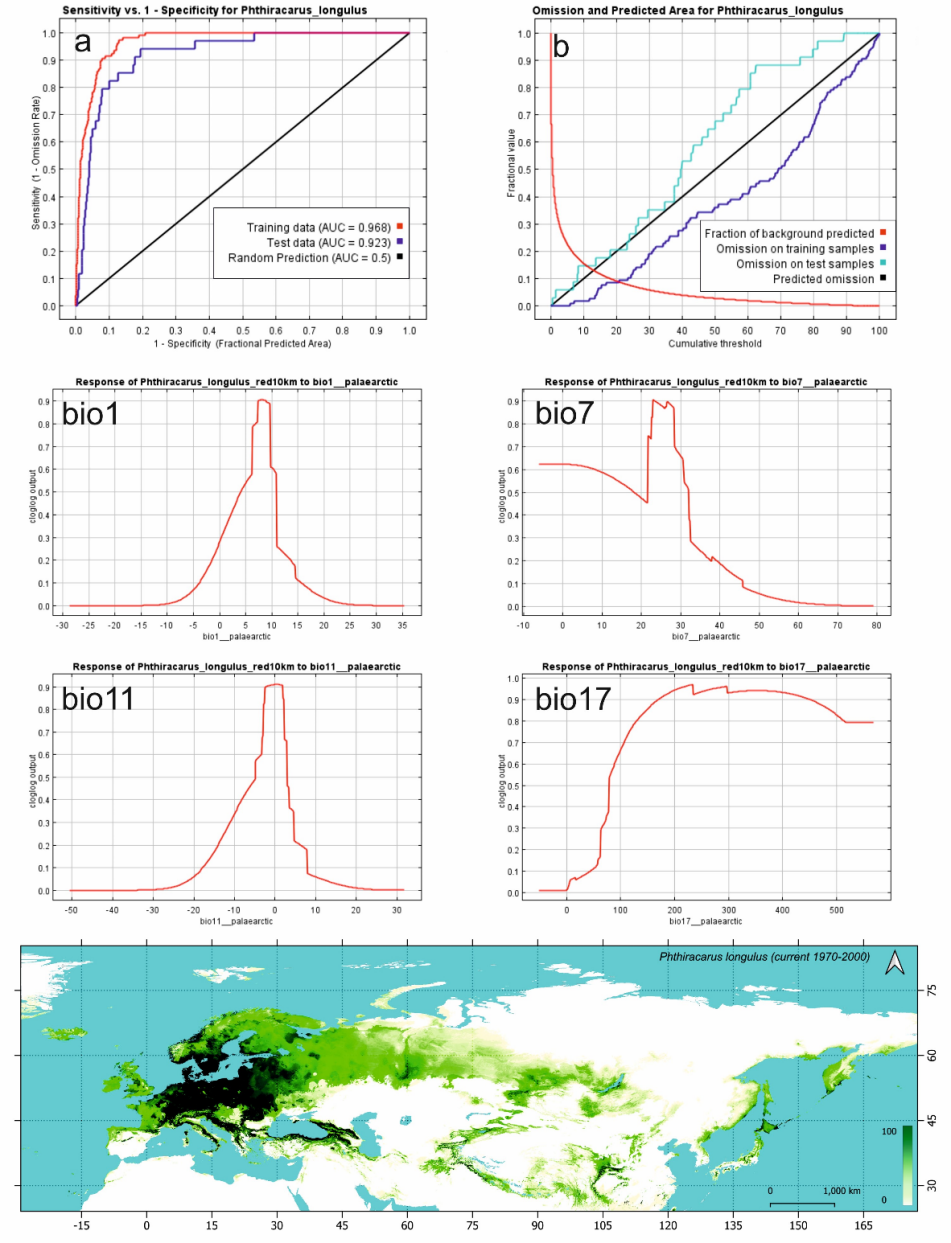
Current model of *Phthiracarus longulus* distribution in the Palearctic: the receiver operating characteristic (ROC) curve (**a**) and test and training omission rate and predicted area as a function of the cumulative threshold curves (**b**) of MAXENT model; response curves of four most important biovariables (bio1, 7, 11, 17; univariate analysis); map of habitat suitability (% scale).

According to ROC, both the training and test AUC’s are above the 0.9 and indicate the excellent accuracy of the model (Fig. 1a). The cumulative threshold chart (Fig. 1b) shows that test and training omission lines are close to the predicted line and the test omission line lies generally above the predicted line that proves the independence of test and training data.

The highest values of contribution rate were found for variables BIO17, BIO7 and BIO5 and reached a total of 52.29% (Table 1). The highest permutation importance was found in BIO7, BIO5 and BIO19 and reached together 53.59%. The highest gain values using only one variable (univariate analysis) were found: in training data for BIO11, BIO1 and BIO6, in test data for BIO12, BIO1 and BIO11 and in AUC for BIO12, BIO11 and BIO1. In case of excluding selected variable (non-specific analysis) the highest decrease in gain were found: in training data for BIO8, BIO15 and BIO3, in test data for BIO9, BIO17 and BIO7 and in AUC for BIO17, BIO15 and BIO9. According to this analysis, the main thermal variables that shape the current distribution of *P. longulus* are BIO7 (Temperature Annual Range), BIO11 (Mean Temperature of Coldest Quarter) and BIO1 (Annual Mean Temperature), while for precipitation variables the most important is BIO17 (Precipitation of Driest Quarter).

**Table 1:** The contribution rate (Cr), permutation importance (Pi), and gain of climatic variables used in MAXENT modelling of *Phthiracarus longulus* distribution.

Variable	Unit	Cr	Pi	TR-excl	TR-uni	TST-excl	TST-uni	AUC-excl	AUC-uni
**BIO1**		**2.787**	**0.097**	**1.9205**	**0.9557**	**1.5570**	**0.9419**	**0.9231**	**0.8568**
BIO2		0.374	2.280	1.9110	0.5702	1.5563	0.3371	0.9233	0.7526
BIO3		5.789	2.945	1.8924	0.6790	1.5476	0.6171	0.9248	0.7875
BIO4		4.696	1.539	1.9078	0.6136	1.5311	0.6299	0.9204	0.7974
BIO5		14.088	13.115	1.9033	0.5407	1.5345	0.5771	0.9211	0.7808
BIO6		0.255	0.033	1.9205	0.9544	1.5343	0.9107	0.9216	0.8525
**BIO7**		**16.078**	**33.997**	**1.9071**	**0.7389**	**1.5176**	**0.6960**	**0.9208**	**0.8114**
BIO8		6.386	6.088	1.8716	0.3959	1.5727	0.2794	0.9243	0.7409
BIO9		2.245	2.564	1.9100	0.8840	1.5036	0.7286	0.9198	0.8191
BIO10		5.963	5.863	1.8930	0.7185	1.6185	0.6525	0.9275	0.8171
**BIO11**		**11.517**	**4.008**	**1.9182**	**1.0195**	**1.5329**	**0.9163**	**0.9219**	**0.8585**
BIO12		0.373	4.545	1.9134	0.7174	1.5334	0.9593	0.9218	0.8654
BIO13		0.381	2.149	1.9166	0.6219	1.5396	0.7923	0.9220	0.8299
BIO14		0.365	1.563	1.9197	0.8906	1.5479	0.5871	0.9231	0.8038
BIO15		1.302	5.956	1.8907	0.4769	1.5245	0.2025	0.9191	0.6884
BIO16		4.315	1.586	1.9119	0.6449	1.5368	0.8307	0.9207	0.8384
**BIO17**		**22.119**	**4.540**	**1.9050**	**0.8908**	**1.5056**	**0.5859**	**0.9183**	**0.8082**
BIO18		0.361	0.650	1.9170	0.4980	1.5564	0.5386	0.9245	0.7861
BIO19		0.609	6.483	1.9040	0.6678	1.5686	0.3807	0.9238	0.7624

The most important variables in bold.

*TR* training gain, *TST* test gain, *AUC* area under ROC (receiver operating characteristic) curve, *excl* gain without variable, *uni* gain with only one variable.

The BIO7 response curve shows that the high probability (≥ 50%) of *P. longulus* occurrence is between − 6.1 and 32.1 °C (Fig. 1). For the BIO11, the most suitable range is between − 4.8 and 2.8 °C. BIO1 response curve shows the high probability of species presence between 4.1 and 10.9 °C, while for the most significant precipitation variable (BIO17) the most suitable range for *P. longulus* presence is between 79 mm and 569 mm.

### Current model of *Phthiracarus longulus* distribution (Fig. 1)

The high suitability areas (> 50% of suitability) for *P. longulus* within the Palearctic cover 2.5 M km^2^ and about 3.3 M km^2^ for 25–50% of suitability (Table 2). Over 86% of area with suitability higher than 50% is located within two biomes, i.e. Temperate Broadleaf and Mixed Forests (76.4%, 1.91 M km^2^) and Temperate Conifer Forests (10.4%, 0.26 M km^2^). Within Temperate Broadleaf and Mixed Forests, almost 50% of area with suitability > 50% is located within three ecoregions, i.e. Central European mixed forests (19.3%, with higher probabilities in its western part), Western European broadleaf forests (16%, with higher probabilities in its eastern part) and Sarmatic mixed forests (11.8%, with higher probabilities in its western part; mainly the southern Sweden and western Latvia–Courland). Most of the highly suitable area (> 50% of suitability) in the current distribution model is located at elevations up to 500 m a.s.l. (Table 3).

**Table 2:** Range of *Phthiracarus longulu*s (in M km^2^) in four classes of suitability, modeled with MAXENT method for current and future (using three Shared Socioeconomic Pathways scenarios and four time horizons) climatic conditions.

Period (period/scenario)		Suitability			
		1–25%	26–50%	51–75%	76–100%
CURRENT		23.6	2.34	0.98	1.52
2021–2040	SSP1-2.6	22.8	2.25	1.03	1.11
2041–2060		22.75	2.44	0.8	0.9
2061–2080		23.11	2.24	0.97	0.86
2081–2100		22.65	2.02	0.9	0.79
2021–2040	SSP2-4.5	23.4	2.38	0.89	0.87
2041–2060		23.11	2.24	0.94	0.84
2061–2080		22.22	1.91	0.9	0.79
2081–2100		22.21	2.05	0.86	0.68
2021–2040	SSP5-8.5	22.85	2.22	1.01	1.14
2041–2060		22.49	2.24	0.93	0.78
2061–2080		21.68	1.75	0.69	0.62
2081–2100		19.69	1.63	0.59	0.55

**Table 3:** Range of *Phthiracarus longulu*s (in M km^2^) at specific elevations based on current and three future (2081–2100) Shared Socioeconomic Pathways scenarios modeled with MAXENT method (for the occurrence probability > 50%).

Elevation	Current	2081–2100 scenario		
		SSP1-2.6	SSP2-4.5	SSP5-8.5
up to 500	1.67	1.05	0.92	0.60
501–1000	0.42	0.28	0.25	0.18
1001–1500	0.20	0.14	0.11	0.06
1501–2000	0.11	0.09	0.08	0.03
2001–2500	0.06	0.05	0.05	0.04
2501–3000	0.03	0.03	0.03	0.03
3001–3500	0.01	0.01	0.02	0.02
3501–4000	2.44E−03	0.01	0.01	0.02
4001–4500	2.73E−04	0.01	0.02	0.02
4501–5000	5.50E−05	0.02	0.03	0.07
5001–5500	1.80E−05	4.50E−03	0.01	0.06
5501–6000	3.60E−05	2.91E−03	4.20E−03	0.01
6001–6500	0.00	0.00	0.00	1.69E−04

Other ecoregions with high probability of *P. longulus* occurrence are located to the north and south of the above-mentioned three ecoregions. In the north these are: Baltic mixed forests (all of its area, i.e. eastern part of Jutland and north-western Poland) and European Atlantic mixed forests (mainly in its north-eastern part, i.e. western part of Jutland and northern Germany). In the south, the higher probabilities are within Carpathian montane forests (most of its area with suitability > 75%), Dinaric Mountains mixed forests (most of its area with suitability > 75%) and Alps conifer and mixed forests (higher probabilities pronounced in southern and northern verges when compared to central parts). Areas with high occurrence probabilities in the latter ecoregion are connected with the same areas within the Apenine Peninsula through the north-western parts of Italian sclerophyllous and semi-deciduous forests which cover most of the Italian Peninsula, however, in Italy we found the higher probability of *P. longulus* occurrence within the Apennine deciduous montane forests and South Apennine mixed montane forests.

The higher probabilities of occurrence we have also found along the borders of Pannonian mixed forests (with visibly lower probability within its central part, i.e. Great Hungarian Plain). Clearly higher probability of occurrence is visible along the southern, eastern and north-eastern coasts of the Black Sea (in the north only to the eastern coast of Sea of Azov). These areas consist of three bioregions: southern Euxine-Colchic broadleaf forests and northern Crimean Submediterranean forest complex, connected it the east with the Caucasus mixed forests. The higher probability areas of the Black Sea coast are connected to the western-European part of the species range through Balkan mixed forests and Rhodope montane mixed forests. In the Southern Europe, higher probabilities of the *P. longulus* occurrence have also been found in the Pyrenees conifer and mixed forests, Corsican montane broadleaf and mixed forests, part of Tyrrhenian-Adriatic sclerophyllous and mixed forests (i.e. central part of Sardinia) and Pindus Mountains mixed forests (Greece and Albania). In the Western-European part of Palearctic, with the lower probability (5–50%), the habitat conditions are suitable for *P. longulus* in the area parts of the Iberian Peninsula (Northwest Iberian montane forests, Iberian conifer forests and Cantabrian mixed forests), British Isles and Scandinavia. From the latter region, suitable habitats for *P. longulus* form a north belt eastward, through the Scandinavian and Russian taiga and Urals montane forest and taiga up to the West Siberian taiga and, more to the south, Western Siberian hemiboreal forests and northern parts of Kazakh forest steppe. The probability of occurrence is, in general, higher in two former regions when compared with West Siberian taiga, and the other suitable habitats are more southward in the direction of Baikal through the Sayan montane conifer forests, South Siberian forest steppe and south-eastern border of East Siberian taiga. In the areas of southern coast of Baikal (Trans-Baikal conifer forests), Daurian forest steppe and Selenge-Orkhon forest steppe there are also some habitats suitable for *P. longulus*. These areas connect with the suitable areas of south-eastern Asia, i.e. Central China Loess Plateau mixed forests and south-western Ordos Plateau steppe, through the parts of Mongolian-Manchurian grassland, with the more suitable Qin Ling Mountains deciduous forests, Qionglai-Minshan conifer forests and parts of Daba Mountains evergreen forests and Guizhou Plateau broadleaf and mixed forests. These habitats are connected with the south-European parts of the possible species range through the Tibet, Himalayas and Afghan mountains (Hindu Kush) and further Elburz Range forest steppe and Caspian Hyrcanian mixed forests. Southern and northern possible Asiatic habitats of *P. longulus* are connected through Tian Shan, Emin Valley steppe and Altai. Suitable areas for the studied species are also located along the coasts of the Sea of Japan, Sea of Okhotsk and north-western coast of Bering Sea, including most of Kamchatka, Sakhalin, Kuril Islands and most of Japan Archipelago (Hokkaido deciduous forests as well as Taiheiyo and Nihonkai montane deciduous forests).

### Future models of *Phthiracarus longulus* distribution (Table 4, Figs. 2, 3 and 4, supplementary figs S2.1-3)

#### Change in distribution during the first period (2021–2040)

**Fig. 2:**
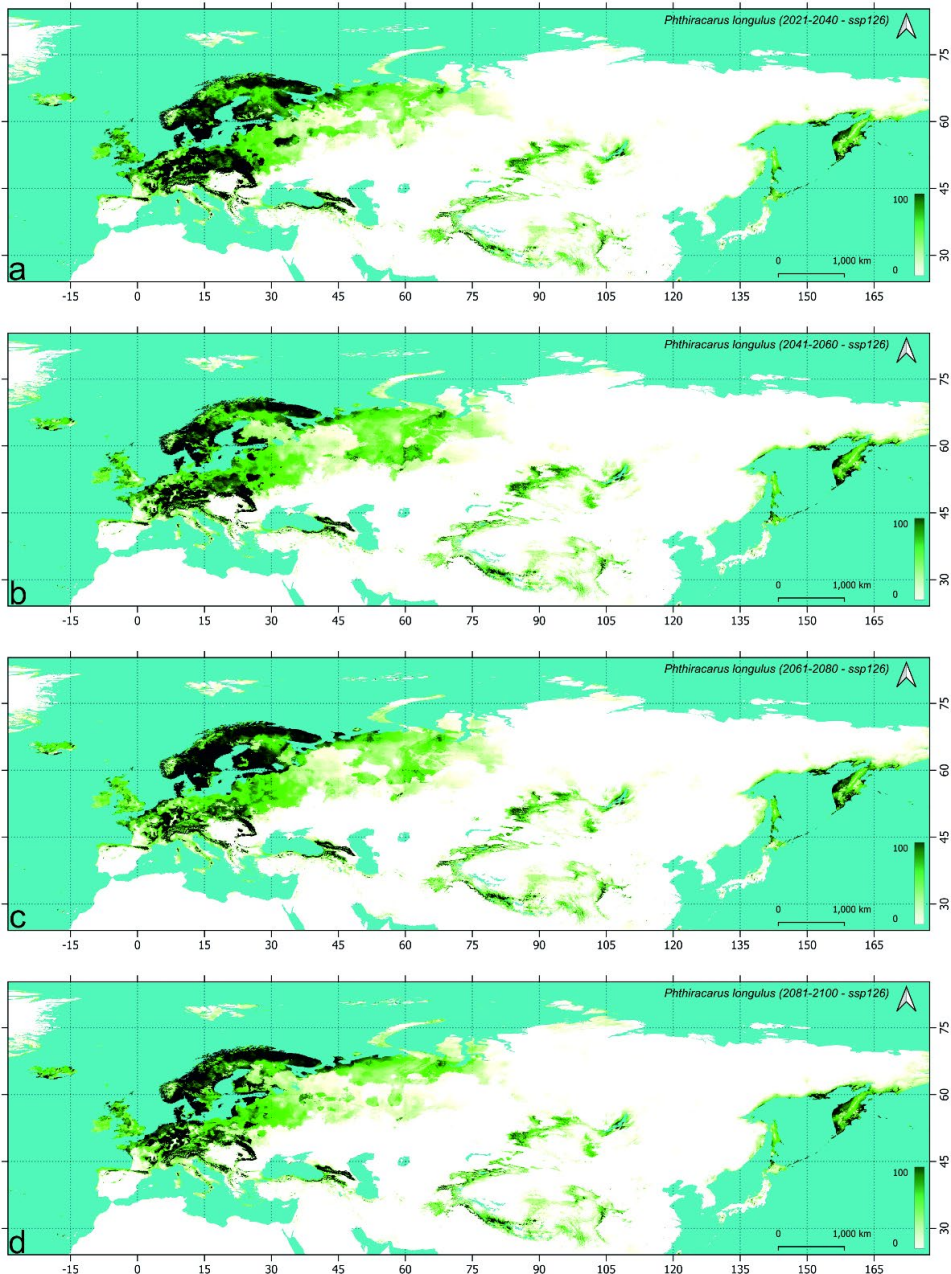
Future models of *Phthiracarus longulus* distribution in the Palearctic under SSP1-2.6 scenario: maps of habitat suitability (% scale) in four time horizons: (**a**) 2021–2040, (**b**) 2041–2060, (**c**) 2061–2080, (**d**) 2081–2100.

In the SSP1-2.6 scenario, the possible range of *P. longulus* is visibly shifted northward (Fig. 2a, S2.1). In the European part of Palearctic, the possibility of occurrence decreases in lowlands and remains high in elevated areas. In the eastern part of Scandinavian and Russian Taiga, two eastward belts (northern and southern) are more suitable for *P. longulus* occurrence. There is also a visible loss of connection between the north-eastern part of Europe with the south-eastern part of West Siberian Taiga, as the northern belt of possible range breaks in its south-eastern part and Kazakh forest steppe. Also, in south-eastern Asia, the possible range of *P. longulus* is much more fragmented. Northward shift of the range is also visible in the eastern verge of Palearctic as the species losses its range in the southern part of Japan Archipelago and western coast of the Sea of Japan. There is also a visible increase in suitability in Kamchatka and the coasts of the Sea of Okhotsk (mainly the Magadan Oblast).

**Fig. 3:**
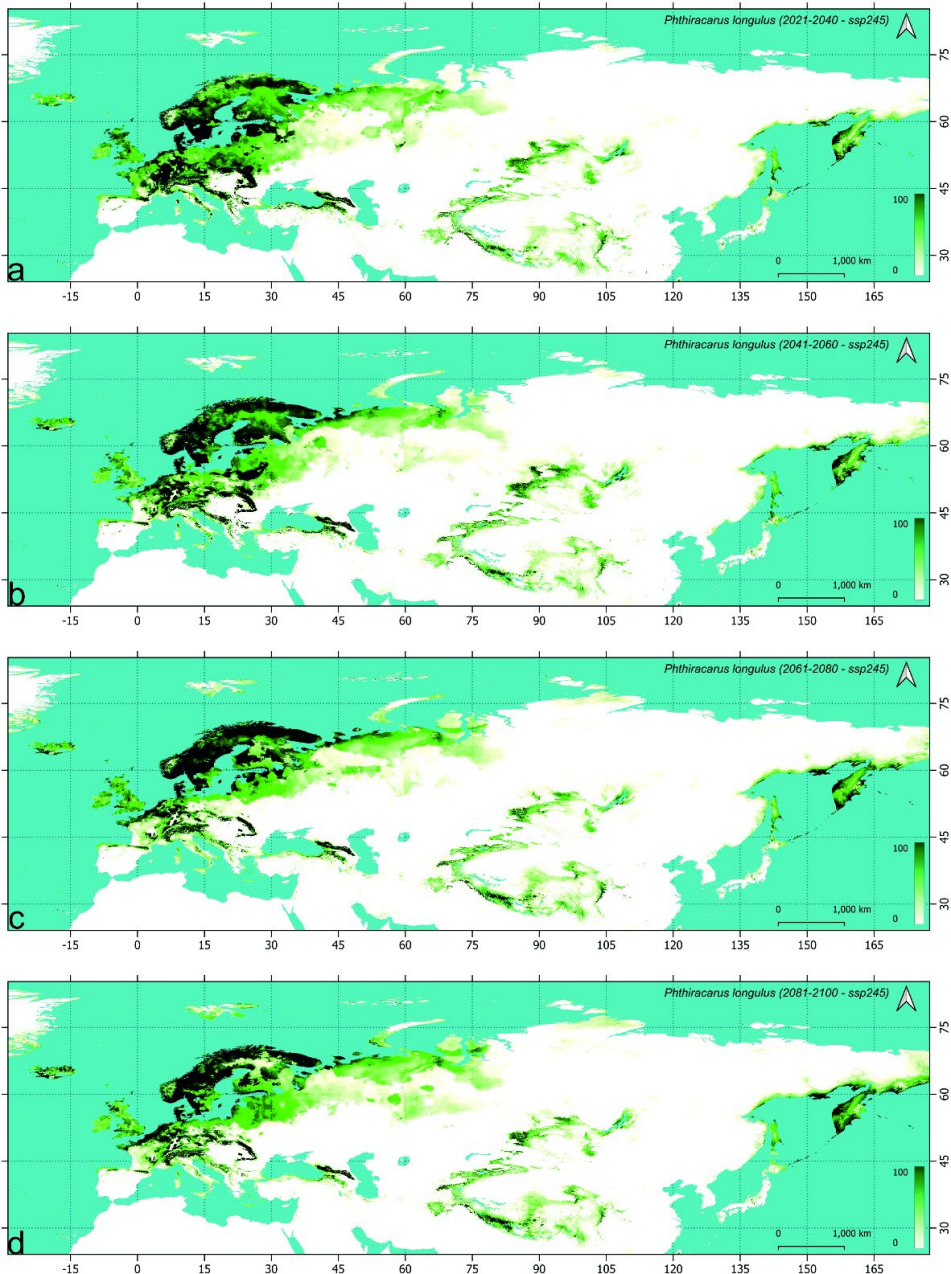
Future models of *Phthiracarus longulus* distribution in the Palearctic under SSP2-4.5 scenario: maps of habitat suitability (% scale) in four time horizons: (**a**) 2021–2040, (**b**) 2041–2060, (**c**) 2061–2080, (**d**) 2081–2100.

**Fig. 4:**
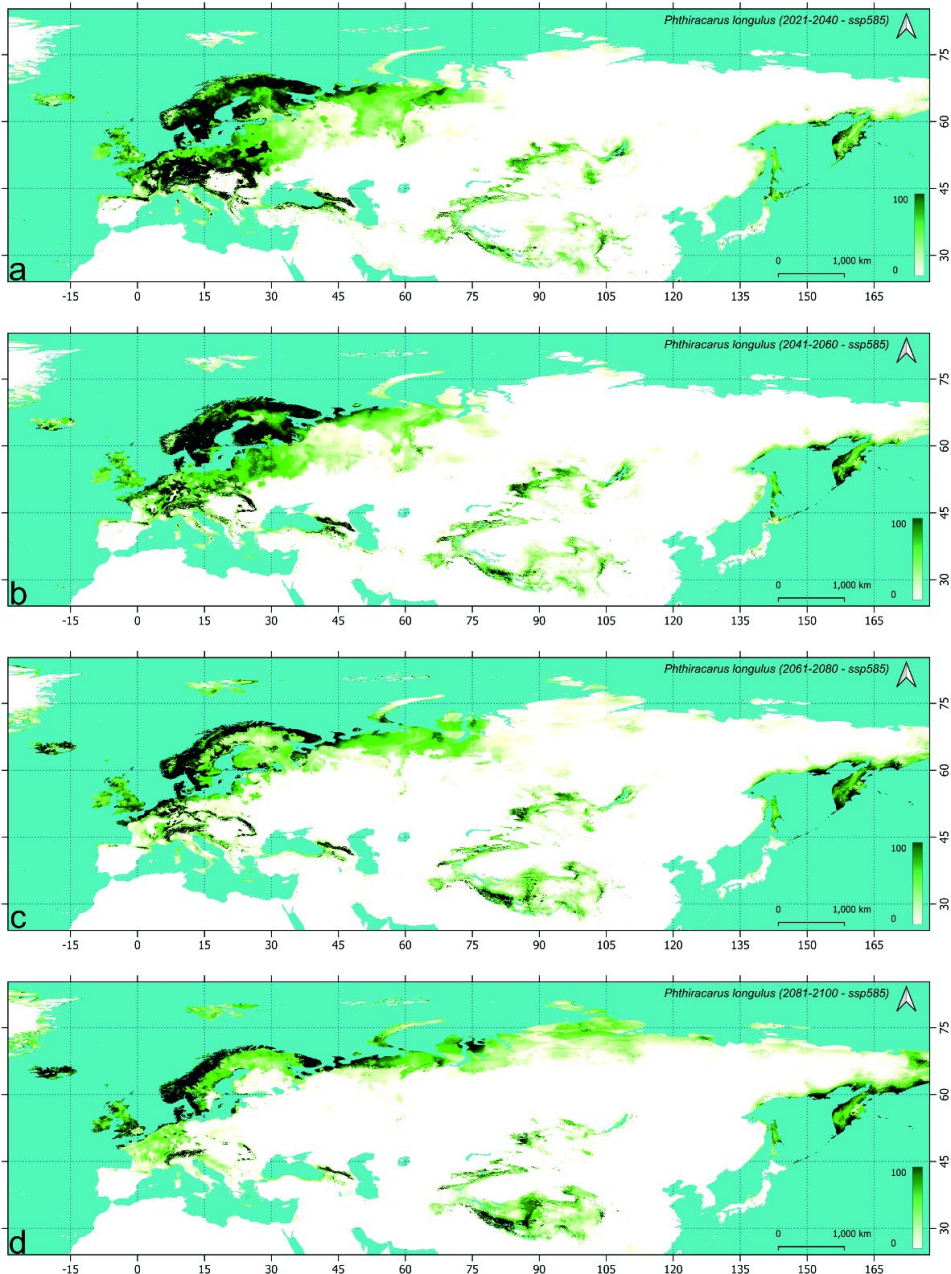
Future models of *Phthiracarus longulus* distribution in the Palearctic under SSP5-8.5 scenario: maps of habitat suitability (% scale) in four time horizons: (**a**) 2021–2040, (**b**) 2041–2060, (**c**) 2061–2080, (**d**) 2081–2100.

When compared to this scenario, the main difference visible in SSP2-4.5 (Fig. 3a, S2.2) is the loss of more suitable areas along the southern coast of the Baltic Sea, the loss of southern more suitable belt of the eastern part of the Scandinavian and Russian Taiga and, at the eastern verge of Palearctic, the possible retracting from the most of Hokkaido. For the worst scenario during this period (SSP5-8.5, Fig. 4a, S2.3) the loss of more areas of the Baltic Sea coast (coast of Gulf of Finland) has been modelled, with visible increase in suitability in northern Scandinavia. The probability of occurrence in the eastern part of Western European broadleaf forests and central part of Central European mixed forests is more similar in SSP5-8.5 and SSP1-2.6 when compared to SSP2-4.5 which is also true for the coast of the Sea of Okhotsk.

The analysis constrained to areas of suitability above 50% shows no remarkable change of distribution in the south-eastern and eastern part of Palearctic with clearly visible changes in its European part. The highest negative balance (expansion minus contraction) and the lowest retained area of the *P. longulus* distribution for the areas of habitat suitability more than 50% for the period 2021–2040 has been recorded in the SSP2-4.5 scenario (Table 4). In the most optimistic and the most pessimistic scenarios, the overall balance has been similar, which resulted in the loss of about 14% of area (> 50% of suitability) while in the intermediate scenario it was about twice higher.

**Table 4:** *Phthiracarus longulus* range (in M km^2^) expansion, contraction, balance (expansion–contraction), and preservation according to step-by-step analysis of future (using three Shared Socioeconomic Pathways scenarios) climatic conditions, using range modeled with MAXENT method (for the habitat suitability > 50%).

Period		Scenario	Species range					
Initial	Final		Expanded	Contracted	Balance	Preserved	Initial	Final
Current	2021–2040	SSP1-2.6	0.76	1.12	− 0.36	1.38	2.5	2.14
2021–2040	2041–2060		0.33	0.77	− 0.44	1.37	2.14	1.7
2041–2060	2061–2080		0.57	0.44	0.13	1.26	1.7	1.83
2061–2080	2081–2100		0.4	0.54	− 0.14	1.29	1.83	1.69
Current	2021–2040	SSP2-4.5	0.57	1.31	− 0.74	1.19	2.5	1.76
2021–2040	2041–2060		0.64	0.62	0.02	1.14	1.76	1.78
2041–2060	2061–2080		0.52	0.61	− 0.09	1.17	1.78	1.69
2061–2080	2081–2100		0.31	0.46	− 0.15	1.23	1.69	1.54
Current	2021–2040	SSP5-8.5	0.84	1.19	− 0.35	1.31	2.5	2.15
2021–2040	2041–2060		0.57	1.01	− 0.44	1.14	2.15	1.71
2041–2060	2061–2080		0.42	0.82	− 0.4	0.89	1.71	1.31
2061–2080	2081–2100		0.39	0.56	− 0.17	0.75	1.31	1.14
Current	2081–2100	SSP1-2.6	0.81	1.62	− 0.81	0.88	2.5	1.69
Current	2081–2100	SSP2-4.5	0.96	1.92	− 0.96	0.58	2.5	1.54
Current	2081–2100	SSP5-8.5	0.9	2.26	− 1.36	0.24	2.5	1.14

#### Change in distribution during the second period (2041–2060)

In the optimistic scenario, the following loss of suitable habitats is visible in the European lowlands but also some higher elevation areas of the European mountain ranges. The main difference, when compared with previous period in this scenario (SSP1-2.6, Fig. 2b, S2.1), is the lowering of suitability on the southern coast of the Baltic Sea and the southern Finland (except coast). Also, the gap in suitable areas is formed between the eastern part of the Scandinavian and Russian taiga and the Scandinavian Peninsula. No remarkable change is visible in the Asiatic part of Palearctic with except the western part of the West Siberian taiga. Clear northward shift is visible in the SSP2-4.5 scenario (Fig. 3b, S2.2), with distinct fragmentation of high suitability areas of the Southern Europe and gathering more suitable areas in Iceland. This trend is also visible in the pessimistic scenario (SSP5-8.5, Fig. 4b, S2.3), with clear loss of suitable areas in European mountain ranges (except higher elevations) and southern coast of the Black Sea.

When the high suitability (> 50%) habitats are considered, the largest range is gained by the *P. longulus* in the intermediate scenario (SSP2-4.5) which is the only with positive area balance (Table 4). In the optimistic and pessimistic scenarios (SSP1-2.6 and SSP5-8.5 respectively), the balance is the same (− 0.44 M km^2^) and despite the higher loss in the pessimistic scenario the final species range is the same in SSP5-8.5 and SSP1-2.6. Consequently, in the second period we observed the preservation of inhabited area in the intermediate scenario and further and similar loss in other scenarios when compared to the first period.

#### Change in distribution during the third (2061–2080) and fourth (2081–2100) periods

During the third period, as observed earlier, the most pronounced change in distribution of *P. longulus* has been modelled in the European part of Palearctic. Apart from the following northward shift, the main difference between optimistic (SSP1-2.6, Fig. 2c, S2.1) and intermediate (SSP2-4.5, Fig. 3c, S2.2) scenarios during 2061–2080 is the considerable loss of suitable areas in the Central European mixed forest and the consequent fragmentation of habitats within Southern Europe in the latter scenario. In the SSP5-8.5 scenario (Fig. 4c, S2.3), the fragmentation is much stronger and the loss of suitable areas is clearly visible also in central and east Scandinavia (Finland and most of Sweden). At the same time, the increasing of suitable areas in the British Isles and Iceland has been modelled. During the fourth period (2081–2100) deeper processes of the northward distribution shift are visible in Europe with clear increasing of habitat suitability along the north-eastern coast of Palearctic, i.e. from the Yamal to Taymyr peninsulas and further to Yakutia and Chukchi Peninsula (Figs.2d, 3d, 4d).

The balance of species ranges during the last period (2081–2100) is negative and similar in the all analyzed scenarios; however, the retained area decreases from the optimistic towards the pessimistic scenario, which results in decreasing of the species range from 1.69 M km^2^ in SSP1-2.6 to 1.14 M km^2^ in SSP5-8.5 (Table 4).

Overally the range of *P. longulus* decreases, in the farthest analyzed time horizon, depending on the scenario: by 32% in SSP1-2.6, 38% in SSP2-4.5 and 54% in SSP5-8.5. In the farthest time horizon, the most stable areas of *P. longulus* distribution are the Jutland with surrounding southern coasts of Scandinavia, islands of the Danish Straits and the region of Trondheim Fjord. In the pessimistic scenario, within the European part of Palearctic the studied species will survive only in the Alps, part of the Carpathians and, more to the south-east, the Caucasus. In the Central Asiatic part of Palearctic the suitable areas for *P. longulus* will remain in the Tibet, Tian Shan, Altai and Sayan mountain ranges. At the eastern verge of Palearctic, the suitable areas for the studied species populations have been modelled within Kamchatka, north-western coast of the Sea of Okhotsk and north-western coast of the Bering Sea.

## Discussion

According to the current 6th Assessment Report (AR6) of the Intergovernmental Panel on Climate Change (IPCC), the human-related global warming and its effects has been strengthened since the AR5 published in 2014^1^. The most prominent hot-spot areas worldwide regarding climatic change are the Mediterranean and North Eastern Europe, and the main emerging hot-spots within Palearctic are Northern Europe, Northern Asia and Greenland at high latitudes and Central Asia and Tibetan Plateau at mid-latitude^33–38^. Observed species range shifts and changes in phenology, for most regions of the world, are attributed to climate change with high or very high confidence^1^. The climate-mediated habitat losses may threaten the long-term chance of species survival as the climate change will potentially overwhelm the capacity for species adaptations and therefore will lead to increase in extinction risk^39,40^. Climate change can influence on animals directly and indirectly. Detailed case-studies on direct and indirect impact of the climate change on wildlife can be found in Green et al.^41^ and particularly for the soil environment in Coyle et al.^22^.

Harvey et al.^16^ emphasized that insects, referred by the authors as central components of many ecosystems, are among the groups mostly affected by the global climate change. Indeed, the insect diversity and impact are crucial for maintaining the life processes (through e.g. pollination), however, mites are at least as much important, contributing to a high extent to matter and energy circulation in terrestrial ecosystems. The most (> 90%) of terrestrial net primary production is finally deposited in the soil (a subsystem of the terrestrial ecosystem) and then undergoes the processes of decomposition; the mineralization is possible thanks to bacteria and fungi that account for 80–90% of the total decomposer biomass and respiration^18,27–30,42,43^. Arthropod mesofaunal groups inhabiting soils are collembolans and mites, and among mites the Oribatida is often the most numerous group in temperate and subtropical forests^44^. Most of the oribatid mites feed on the surface of decaying organic matter while e.g. immature Phthiracaroidea burrow into substrate to use the underlying resources^31^, contributing therefore to a high extent to decomposition. The soil animals also improve the dispersal abilities of fungi and bacteria, as these groups can be transported on the body surface and also in the digestive tract and dispersed with fecal pellets. Dispersal of ectomycorrhizal propagules by mites can moreover influence on the fungi-plant symbiosis, with its effects in the above-ground part of the ecosystem, and some oribatids have a strong “pollinator-like” relationship with mosses^31,44^. Therefore, the climatically driven destabilization of ecosystems and the consequent disturbance of the provisioning of ecosystem services will be in our opinion much more severe than predicted by Harvey et al.^16^.

Hill et al.^9^ used Lepidoptera as a model group to perform analysis of possible responses to climate change, considered four groups of different-level responses: organismal, populational, phenological and community-level effects, and discussed the range expansion and contraction phenomena, paying attention to e.g. the altitudinal and latitudinal shifts, and possibility of habitat fragmentation.

The temperature in e.g. European Alps is increasing at a rate of about 0.36 °C per decade since 70s^45^. The increasing temperature leads to isothermal elevational shift and the upslope shift of optimum elevation and the upper range limit of many taxa. In fact, the European Alps didn’t experience such climatic change from millennia; the extent of current changes is, however, likely to be underestimated^10,45,46^. Simultaneously, the general predictions of Ohlemüller et al.^47^ show the average northward shift of European climatic zones from 272 to 645 km up to 2100 depending on the climatic scenario. This also entails the increase in altitudinal range margins of plants, animals and fungi in Scandes, Alps and Carpathians with strong predictions of the loss of species richness in Mediterranean and Atlantic regions^45,48–52^.

The phenomena of latitudinal/elevational shifts are well documented and widely discussed. Wiens^3^ presented many examples of the modern climate change that caused the local extinction at the “warm edges” (lower altitudes and latitudes) of species’ ranges and simultaneous “cold-edge” range expansion, with elevational range shift in plants and elevational/latitudinal shifts in animals. Hickling et al.^53^ recorded this phenomenon in most of the studied species, however, for some taxa also the reverse trend or no change was observed. Also, Wilson et al.^39^, Gallant et al.^54^ and D’Orangeville et al.^55^ discussed many examples of the expanding the distribution to higher latitudes and, in southern areas, to higher elevations. For the most of the butterfly species studied by Wilson et al.^39^ the impact of climate change on optimal elevation further implied the reduction of habitable area by one-third in 30 years.

Although the *P. longulus* is considered the Holarctic species, within Palearctic the most suitable areas were modelled mainly in Europe. The future scenario models of *P. longulus* distribution show two clearly visible, and interconnected, processes: the northward shifts of suitable areas and the habitat fragmentation, particularly emphasized in the upslope shifts in the Southwestern-European mountain systems.

The impact of global climatic changes on the particular species can also spread further on other related taxa and result in e.g. decoupling from resources and predators or increase the competition as found in e.g. *Vulpes vulpes* and *V. lagopus* relationship and some predatory birds^9,54,56,57^. Arthropods that are primary consumers are probably less dependent on fluctuations of feeding resources if compared with climatic variability^58^. Oribatid mites feed primarily on dead organic matter and will directly track the suitable climate, but also habitats rich in preferred substrates. The juveniles of widely studied *Steganacarus magnus*^59^ develop inside tissues of the fallen cones of *Pinus sylvestris* Linnaeus, 1753^60–63^ so it is expected that this species will track the distribution of the Scots Pine. Changes in tree/fungal distributions are of great importance in the discussion on driver-factors of the future distributions of primary consumers like Phthiracaridae. It could be imagined that the diet of these mites will change due to climatic pressure. The gut microbiota (poorly studied in mites, e.g. in *S. magnus* by Webb^61^) is essential in digestion; the influence of climate change on this part of animal physiology was reviewed by Williams et al.^64^.

The concept of “northern biodiversity paradox”, proposed by Berteaux et al.^65,66^, suggests that the climate change can lead to potential biodiversity increase in northern regions, as some species at their northern limit will shift poleward when tracking suitable climates. The overall biodiversity, however, will decrease as many species become trapped when coping with climate change e.g. taxa of islands, peninsulas or mountaintops that will not be able to extend their range poleward or shift to higher elevations, as found in e.g. land snail *Arianta arbustorum*^3,67,68^. Because of the environmental gradient, also e.g. oxygen tension (altitudinally) or UV intensity (altitudinally and latitudinally) can cause some negative effects, depending on the e.g. role of oxygen in thermal limitation and regulation of melanization to prevent the impact of harmful part of sun radiation and overheating^69–72^.

In the case of *P. longulus* the “southern biodiversity paradox” can be driven by the elevational shift in Southern Europe which will result in habitat fragmentation. Some papers list the positive effects of the habitat fragmentation that cannot be excluded, but are usually masked by its negative effects^73,74^. Nevertheless, the climate-driven habitat fragmentation can promote allopatric speciation and species diversification, as found between Pleistocene climatic oscillations and genus *Sclomina* (Hemiptera: Reduviidae)^75^. If the local populations of *P. longulus* in the south-European mountain systems will survive for the enough time, the chance that they evolve into distinct species will raise. The negative effect of fragmentation is the increasing rate of inbreeding, however also outbreeding can lead to depression and can cause extinction in particular cases^16,76,77^. At present, it is hard to conclude if the elevational shift of *P. longulus* populations in Southern Europe along with the latitudinal northward shift can result in either positive or negative effects.

Willis et al.^78^ and Vitasse et al.^45^ showed that the rate of colonization doesn’t equally follow the climate change, i.e. the species disperse more slowly than the climate change could allow, causing the disequilibrium between species’ and climate distributions.

The dispersal capacity of oribatid mites is generally poor and phoresy is rare in this suborder^79^. Therefore, it is unclear if the *P. longuls* will be able to follow the shift of climate to disperse poleward, especially to reach the north-Asiatic part of Palearctic. It depends on the phenotypic plasticity as well individual physiological and behavioral responses regarding the climate change^9,16,80–82^. Nevertheless, in our models, some areas seem to provide stable environments for *P. longulus* up to year 2100 regardless of the climatic scenario (Jutland, southern coasts of Scandinavia, Danish Strait, Trondheim Fjord). Therefore, we suppose that the extinction of *P. longulus* due to climate change is unlikely.

## Conclusions and further implications

Our model of the *P. longulus* response is based on macroclimatic predictions. According to the recently published article of Lembrechts^83^ it cannot be excluded that many species will achieve the reproductive success under unsuitable macroclimate if they will find proper microclimates, as those two views that differ in resolution can differ vastly from the viewpoint of habitat suitability. According to this author, this methodological discrepancy can explain the divergence between observed shifts in climate and species distributions. The problem is that the macroclimate and microclimate are main and direct, but not the only, factors that driver the distributions. Even one of the other, non-climatic factors that rely more on macroclimate than microclimate (e.g. shifts of plants that are preferred feeding resources) can alter the distribution regardless of microclimate. The widely distributed and relatively slow-moving, non-phoretic *Phthiracarus longulus* should be further studied in details regarding its actual distribution, physiology, phenology, dispersal ability, population genetic structure and phenotypic plasticity to evaluate our climatic model and explore all other factors that shape the soil fauna in a face of the global change. Our models could help with planning future investigation areas to pinpoint the current distribution borders of *P. longulus*. It will also help with the evaluation of the distribution shift shown in our models, depending on the climatic scenario that we will follow in the future. Modelled change of distribution in the area of the southern and eastern coasts of the Baltic Sea, in our opinion, justifies the selection of this region as the future monitoring site of distribution change of *P. longulus*. Our models would also be helpful for further distribution dynamics studies within *Phthiracarus* and other genera of ptyctimous mites.

## Materials and methods

### The studied species

*Phthiracarus longulus* (Koch)^32^ is a member of chelicerate superorder Acariformes, order Sarcoptiformes, suborder Oribatida Dugès, 1834^84^ infraorder Mixonomata Grandjean, 1969^85^, superfamily Phthiracaroidea Perty, 1841^86^ and family Phthiracaridae Perty, 1841^86^. According to Niedbała and Liu^87^ the family Phthiracaridae is monotypic and the genus *Phthiracarus* Perty, 1841^86^, comprised of 153 species, is cosmopolitan (except Antarctica). *Phthiracarus longulus* is a Holarctic, Panpalearctic species also found in Uruguay (probably introduced)^87,88^. Mites from superfamilies Phthiracaroidea and Euphthiracaroidea are ptychoid and sometimes grouped under the name Euptyctima Grandjean, 1967^89^. Immature phthiracaroid mites burrow in decomposing twigs and leaf-tissues and feed on microbial and fungal decomposers and also decaying tissues. Phthiracaroid females retain eggs until embryogenesis completion and deposit prelarvae. Juveniles are endophagous and are rarely found outside self-buried corridors in which they live^31^. We selected *Phthiracarus longulus* for this first-ever modelling of mite distribution using the maximum entropy (MAXENT) method because of the species commonness within studied samples. Based on Niedbała^88^ and our new data on distribution of ptyctimous mites, *Phthiracarus longulus* is the most frequent in locations (about 24%) and the most abundant (48% of locations with dominance 50% or more, 32% of locations with only *P. longulus*) within genus.

### Modelling methods and data

Overally, for *Phthiracarus longulus* we collected 282 presence points, including 247 points from Niedbała^88^ and 35 new records (see Annex – Supplementary 1). Records were checked and cross-validated with maps.google.com and mapy.cz. Other presence points coordinates were searched using record descriptions with online tools and were specified with maximum possible accuracy. Altogether 230 presence points were recognized as correctly geolocated and further processed using QGIS 3.18.3-Zürich with GRASS 7.8.5. To reduce sampling bias, the occurrence points were filtered using a distance of 10 km. Finally, the 139 presence points were used in the Environmental Niche Modelling (ENM) using the maximum entropy (MAXENT) method.

For the current distribution modelling we used the all 19 bioclimatic variables (2.5 arc-min spatial resolution, averaged 1970–2000) from WorldClim 2.1 database (www.worldclim.org^90^). For the future projections of *P. longulus* distribution we used the same database for four future periods (2021–2040, 2041–2060, 2061–2080 and 2081–2100) and three Shared Socio-economic Pathways (SSPs) models: SSP1-2.6, SSP2-4.5 and SSP5-8.5^1^. Each biovariable was cropped to Palearctic. All macroclimatic models of *P. longulus* distribution were prepared using maximum entropy modelling with MAXENT 3.4.4 software^91,92^. We used the auto-features, Cloglog output, 25% of presence records used for testing, jackknife analysis of variable importance, regularization multiplier 1 and 500 iterations. Analysis of current model of distribution in ecoregions was based on Ecoregions 2017©^Resolve^ shapefile (ecoregions.appspot.com^93^).

## Electronic supplementary material

Below is the link to the electronic supplementary material.

Supplementary Material 1.

## Data Availability

Data is provided within the manuscript or supplementary information files.
