# Supplementary material for: Distribution of euptyctimous mite Phthiracarus longulus (Acari: Oribatida) under future climate change in the Palearctic
Source: Sci Rep. 2024 Sep 19;14:21913. doi: 10.1038/s41598-024-72852-8 (PMC11412984; doi:10.1038/s41598-024-72852-8)
Supplement: Supplementary file 1 — Supplementary Material 1. [file 41598_2024_72852_MOESM1_ESM.pdf]

Supplementary 1 Distribution of euptyctimous mite *Phthiracarus longulus* (Acari: Oribatida) under future climate change in the Palearctic, Scientific Reports

Annex – list of *Phthiracarus longulus* new localities (number of individuals after dash at the end, samples comma-separated)

1. **Belgium**, Brussels, Forest park (Bois de la Cambre Terkamerenbos), 50°47′54.9″N, 4°23′18.9″E, 116 m a.s.l., leg J. Błoszyk, 02.08.2020, very old beech trees, elms, maple trees, undergrowth scanty, dry, near an old ash tree, sieved material – 1.
2. **Belgium**, Brussels, Forest park (Bois de la Cambre Terkamerenbos), 50°47′54.9″N, 4°23′18.9″E, 116 m a.s.l., leg J. Błoszyk, 02.08.2020, deciduous forest with oak-hornbeam forest trees, rotten wood from a lying trunk of a birch tree; ø 30 cm, length 1 m, moderately rotten, damp rotten wood – 1.
3. **Belgium**, Brussels, Forest park (Bois de la Cambre Terkamerenbos), 50°47′57″N, 4°23′23″E, 89 m a.s.l., leg J. Błoszyk, 02.08.2020, beech tree stand, approx. 50 years old, undergrowth scanty, sieved litter – 1 (new for Belgium).
4. **France**, near Bena, the car park near the highway, alluvial forest, 46°18′37″N, 5°15′37″E, leg. J. Błoszyk, 27.09.2019, a rotten oak tree stump – 1,
5. **France**, near Bena, the car park near the highway, alluvial forest, 46°18′37″N, 5°15′37″E, leg. J. Błoszyk, 27.09.2019, tree trunk moss – 2.
6. **Georgia**, Greater Caucasus, Svanetia, on the trail leading to the monastery, E of Akhalsheni village (near the Mazeri village), 43°4′31.072″N, 42°38′1.928″E, 1671 m a.s.l., leg. M. Podsiedlik, 25.05.2018, decaying wood of *Fagus orientalis* – 3.
7. **Germany**, easten part, Bastei Gebiet rocks, leg. W. Niedbała, 19.09.2016, liter from beech forest – 12.
8. **Germany,** Schonow (Bernau bei Berlin), Schonwalder Chaussee, Brandenburg, leg. T. Rutkowski & M. Zacharyasiewicz, 01.08.2019, litter under pines – 3, 9, 5, 1,
9. **Germany,** Schonow (Bernau bei Berlin), Schonwalder Chaussee, Brandenburg, leg. T. Rutkowski & M. Zacharyasiewicz, 01.08.2019, litter under lime trees – 1, 6, 1,
10. **Germany,** Schonow (Bernau bei Berlin), Schonwalder Chaussee, Brandenburg, leg. T. Rutkowski & M. Zacharyasiewicz, 01.08.2019, litter under pines and oaks – 3,
11. **Germany,** Schonow (Bernau bei Berlin), Schonwalder Chaussee, Brandenburg, leg. T. Rutkowski & M. Zacharyasiewicz, 01.08.2019, litter under oaks – 2, 4.
12. **Italy**, Trento province, Dolomites, Canazei, 1 600 m a.s.l., leg. W. Niedbała, 06.10.2017, liter in the fir forest – 24.
13. **Kyrgyzstan**, the alpine Ala Archa NP in the Tian Shan mountains, leg. W. Niedbała, 07.10.2019, litter from various species of deciduous and coniferous (cyprus) trees – 17.
14. **Norway**, Bergen, Espegrend Marine Research Field Station, suburban forest area, Scots pine, 60°16′10.3″N, 5°13′20.9″E, 3 m a.s.l., leg. S. Kaczmarek, 25.04.2018, moss on the rock, humid – 8.
15. **Norway**, Bergen, Espegrend Marine Research Field Station, suburban forest area, Scots pine, 60°16′10.1″N, 5°13′18.9″E, 4 m a.s.l., leg. S. Kaczmarek, 25.04.2018, moss from a cut pine trunk, approx. 1 m from the soil surface, humid – 9.
16. **Norway**, Bergen, Espegrend Marine Research Field Station, suburban forest area, Scots pine, 60°16′9″N, 5°13′17.7″E, 3 m a.s.l., leg. S. Kaczmarek, 25.04.2018, moss on the rock, humid – 3.

17. **Norway**, Bergen, Espegrend Marine Research Field Station, suburban forest area, Scots pine, 60°16′9″N, 5°13′21.5″E, 6 m a.s.l., leg. S. Kaczmarek, 25.04.2018, moss on the rock, humid – 4.
18. **Norway**, Bergen, Espegrend Marine Research Field Station, suburban forest area, Scots pine, 60°16′8.1″N, 5°13′20.7″E, 8 m a.s.l., leg. S. Kaczmarek, 25.04.2018, moss on the rock, humid – 1.
19. **Norway**, Hamar, suburban forest area, 60°47′28.7″N, 11°2′26.1″E, 141 m a.s.l., leg. S. Kaczmarek, 25.07.2017, moss growing on the wall, near the lake in the coastal forest belt (Short maple, European ash, birch, nettles), shaded place, humid – 1.
20. **Norway**, Hamar, suburban forest area, 60°47′29.5″N, 11°2′13.6″E, 129 m a.s.l., leg. S. Kaczmarek, 25.07.2017, moss on the rock, on a slope near the lake, humid – 1.
21. **Norway**, Hamar, suburban forest area, 60°47′30.4″N, 11°2′19.2″E, 136 m a.s.l., leg. S. Kaczmarek, 25.07.2017, moss on a birch trunk, on a slope towards the lake, humid – 2.
22. **Norway**, Hamar, suburban forest area, 60°47′35.3″N, 11°2′11.4″E, 148 m a.s.l., leg. S. Kaczmarek, 25.07.2017, moss on the rock (birch, rowan, ash), moderately humid – 1.
23. **Russia**, Buryatia Republic, Barguzinsky district, near Lake Baikal, near Maksimiha village, taiga forest, 53°16′10.87″N, 108°47′25.45″E, leg. A.V. Tolstikov, 13.08.2013, mosses and leaf litter – 12.
24. **Russia**, Buryatia Republic, Barguzinsky district, near Lake Baikal, near Maksimiha village, 53°15′50.77″N, 108°48′23.85″E, leg. A.V. Tolstikov, 14.08.2013, mosses on bog – 1.
25. **Russia**, Buryatia Republic, Barguzinsky district, near Lake Baikal, near Shamanka village, taiga forest, 54°28′0.76″N, 110°27′26.88″E, leg. A.V. Tolstikov, 13.08.2013, leaf litter – 16.
26. **Russia**, Buryatia Republic, Barguzinsky district, Ushkanii Islands, 53°51′9″N, 108°36′18.65″E, leg. A.V. Tolstikov, 15.08.2013, leaf litter in forest on Bolshoy Ushkaniy Island – 1.
27. **Russia**, Buryatia Republic, Kurumkansky district, near Lake Baikal, near Adamovo village, taiga forest, 53°29′10.78″N, 109°17′50.31″E, leg. A.V. Tolstikov, 13.08.2013, mosses and leaf litter near small river – 2.
28. **Russia**, Caucasus, Northern Ossetia, Digoriya, Irafsky region, Kubus mountain, 2082 m a.s.l., leg. A.A. Prokin, 09.05.2016, *Sphagnum* spp. – 3.
29. **Russia**, Kamchatka, Elizovo, deciduous forest with dominant birch (*Betula ermanii*) with herbaceous vegetation, 53°10′12.3″N, 158°21′44.6″E, 117 m a.s.l., leg. S. Kaczmarek, 01.07.2014, leaf litter – 1.
30. **Russia**, Kamchatka, Posyolok Zaozjernyj village, about 100 m from the shore of Pacific, forest belt with dominant poplar (*Populus* sp.), 52°59′19.1″N, 158°50′11.9″E, 6.6 m a.s.l., leg. S. Kaczmarek, 03.07.2014, moist poplar leaf litter with *Viola crassa* – 6.
31. **Russia**, Kamchatka, Posyolok Zaozjernyj village, about 100 m from the shore of Pacific, forest belt with dominant of pine (*Pinus pumila*), 52°59′16.9″N, 158°50′17.3″E, 7.5 m a.s.l., leg. S. Kaczmarek, 03.07.2014, moist pine litter with detritus – 3.
32. **Russia**, Kamchatka, Special Protected Area „Nalychevo Nature Park", Valley of Avacha Volcano, deciduous forest with dominant birch (*Betula ermanii*) with addition of alder (*Alnus fruticosa*), 53°13′34.8″N, 158°40′40.7″E, 502 m a.s.l., leg. S. Kaczmarek, 20.06.2014, leaf litter with detritus – 1.

33. **Russia**, Kamchatka, Special Protected Area „Nalychevo Nature Park", Valley of Avacha Volcano, deciduous forest with dominant alder (*Alnus fruticosa*) with addition of birch (*Betula ermanii*), 53°13′59.8″N, 158°40′59.6″E, 548 m a.s.l., leg. S. Kaczmarek, 20.06.2014, leaf litter with detritus – 1.
34. **South Korea**, Jumbong Mountains, mixed forest, 38°00′-38°05′N, 128°20′-128°30′E, leg. S. Kaczmarek, 10.05.1996, moss on the stone – 1 (new for S Korea).
35. **Ukraine**, Karmalyukova Hora Landscape Park, deciduous forest area (ash, hornbeam, maple, oak, hazel, ivy), 48°45′0″N, 26°39′18″E, 320 m a.s.l., leg. S. Kaczmarek, 15.07.2015, moss on the trunk of a lying tree, humid – 1.

Supplementary 2 Distribution of euptyctimous mite *Phthiracarus longulus* (Acari: Oribatida) under future climate change in the Palearctic, Scientific Reports

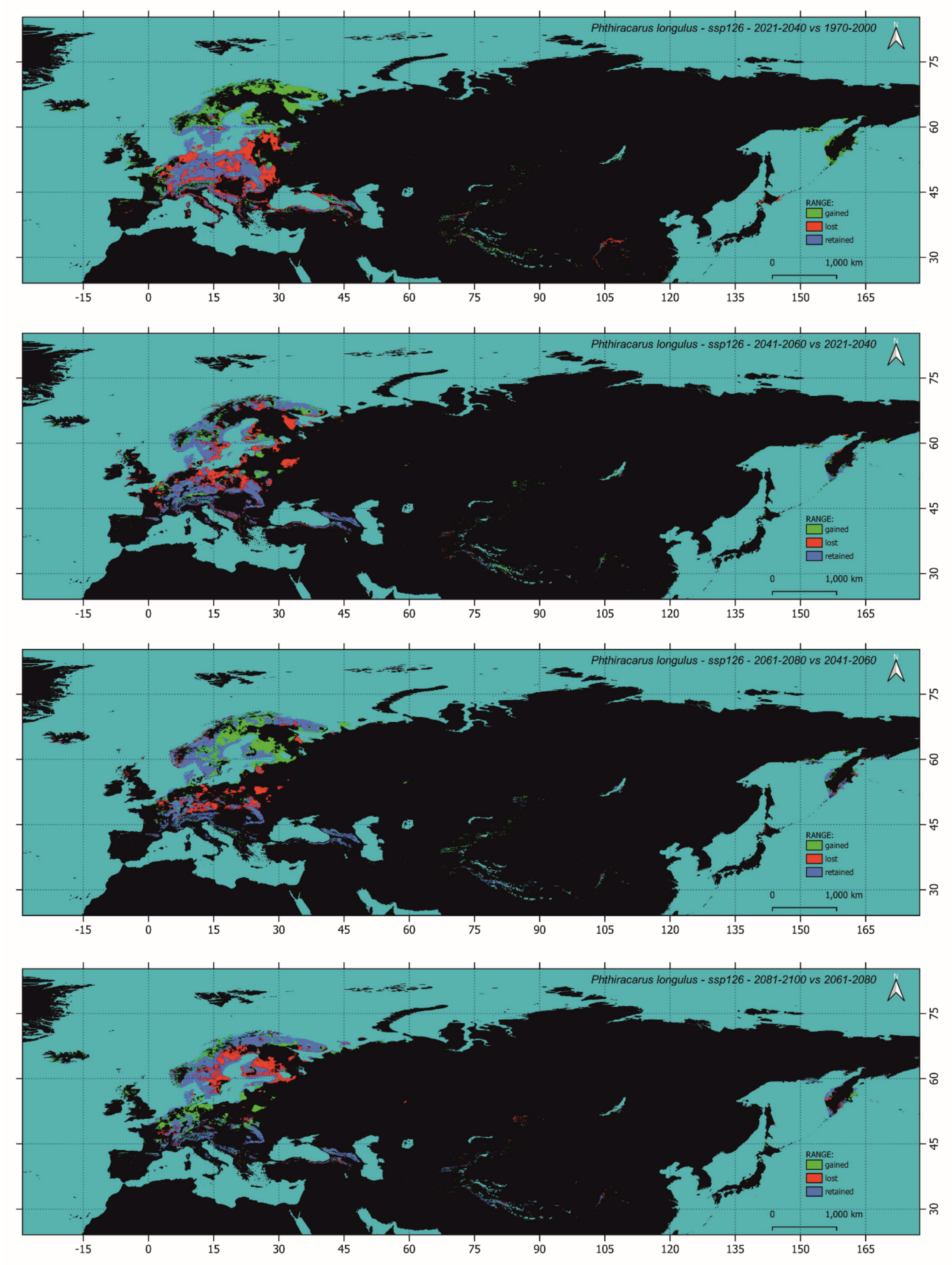

Figure S2.1. Comparison of *Phthiracarus longulus* distribution models using step-by-step procedure (each map shows the range contraction / expansion / preservation of the next period compared with previous period) under SSP1-2.6 scenario

Supplementary 2 Distribution of euptyctimous mite *Phthiracarus longulus* (Acari: Oribatida) under future climate change in the Palearctic, Scientific Reports

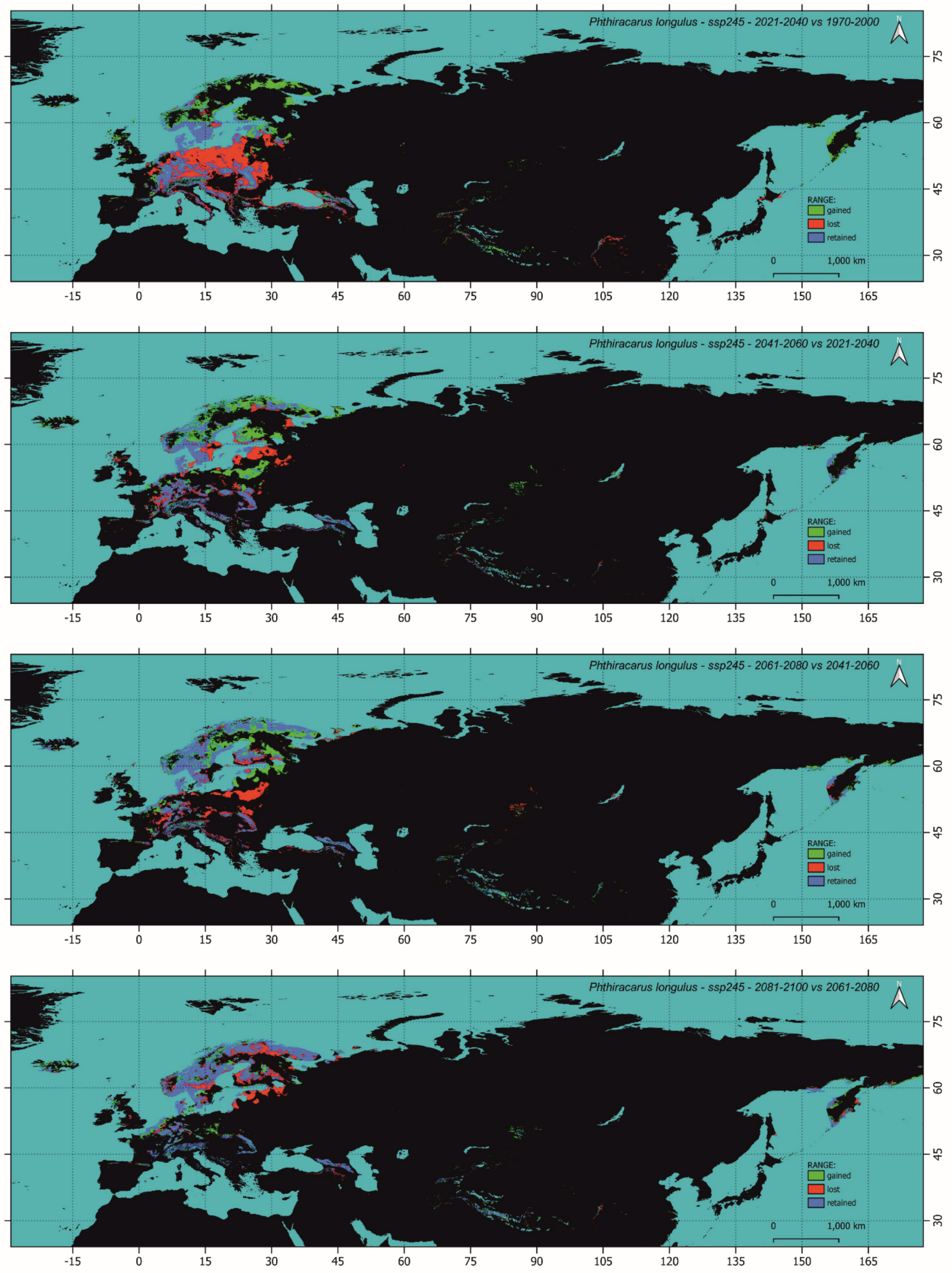

Figure S2.2. Comparison of *Phthiracarus longulus* distribution models using step-by-step procedure (each map shows the range contraction / expansion / preservation of the next period compared with previous period) under SSP2-4.5 scenario

Supplementary 2 Distribution of euptyctimous mite *Phthiracarus longulus* (Acari: Oribatida) under future climate change in the Palearctic, Scientific Reports

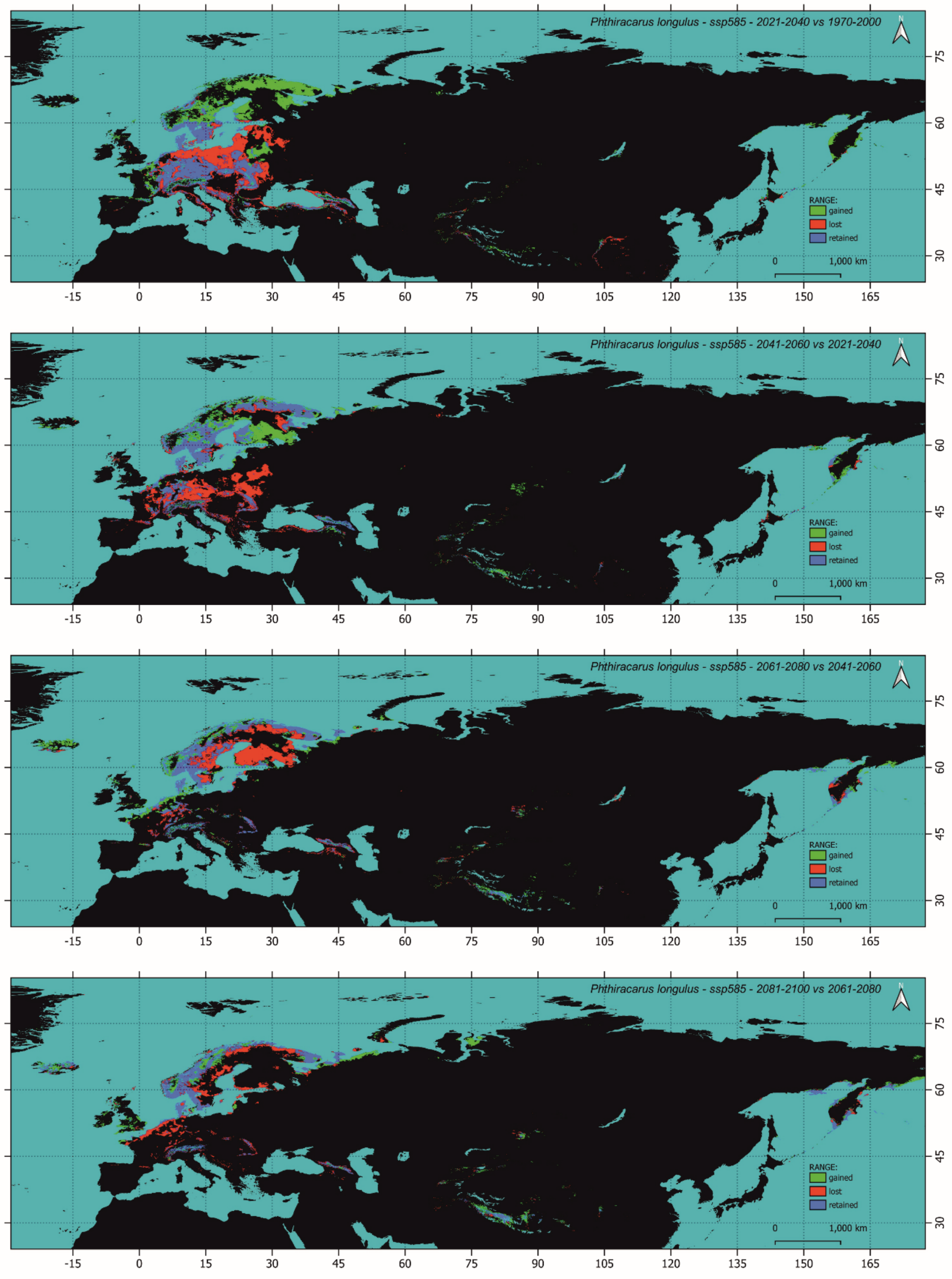

Figure S2.3. Comparison of *Phthiracarus longulus* distribution models using step-by-step procedure (each map shows the range contraction / expansion / preservation of the next period compared with previous period) under SSP5-8.5 scenario
